# Smoking and Radiolucent Periapical Lesions in Root Filled Teeth: Systematic Review and Meta-Analysis

**DOI:** 10.3390/jcm9113506

**Published:** 2020-10-29

**Authors:** Daniel Cabanillas-Balsera, Juan J. Segura-Egea, María Bermudo-Fuenmayor, Jenifer Martín-González, María Carmen Jiménez-Sánchez, Victoria Areal-Quecuty, Benito Sánchez-Domínguez, Paloma Montero-Miralles, Eugenio Velasco-Ortega

**Affiliations:** 1 Department of Stomatology, Section of Endodontics, School of Dentistry, University of Sevilla, 41009 Sevilla, Spain; danielcaba@gmail.com (D.C.-B.); mbfuenmayor@gmail.com (M.B.-F.); jmartin30@us.es (J.M.-G.); v.arealquecuty@hotmail.com (V.A.-Q.); beni2506@yahoo.es (B.S.-D.); montero_paloma@hotmail.com (P.M.-M.); 2 Materials Science Institute of Sevilla (ICMS), Joint CSIC-University of Sevilla Center, 41092 Sevilla, Spain; jimenezsanchez6@gmail.com; 3 Department of Stomatology, Section of Comprehensive Dentistry, School of Dentistry, University of Sevilla, 41009 Sevilla, Spain

**Keywords:** endodontic medicine, persistent apical periodontitis, radiolucent periapical lesion, root canal treatment outcome, root-filled teeth, smoking habits

## Abstract

Aim: This systematic review and meta-analysis aimed to investigate the association between smoking habits and the prevalence of radiolucent periapical lesions (RPLs) in root-filled teeth (RFT). Methods: The Population, Intervention, Comparison, and Outcome (PICO) question was: in adult patients who have RFT, does the absence or presence of a smoking habit affect the prevalence of RPLs associated with RFT? Systematic MEDLINE/PubMed, Wiley Online Database, Web of Science, Scopus, and PRISMA protocol were used to evaluate and present the results. Studies comparing smokers with control non-smoker subjects, including RFT, and providing data on the prevalence of RFT with RPLs, were included. The Grading of Recommendations, Assessment, Development, and Evaluation (GRADE) system was used for certainty in the evidence. The risk of bias was assessed according to Cochrane Collaboration common scheme for bias and ROBINS-I tool. Cumulative meta-analysis was performed with a random effects model. PROSPERO registration code: CRD42020165279. Results: Four studies reported data on inclusion criteria, representing data from 9257 root-filled teeth—4465 from non-smokers and 4792 from smoker patients. The meta-analysis provided an odds ratio indicating a significant association between smoking and higher prevalence of root filled teeth with radiolucent periapical lesions (OR = 1.16; 95% CI = 1.07–1.26; *p* = 0.0004). The certainty of the literature assessment was moderate per GRADE. The ROBINS-I tool classified three studies as low risk of bias, and the fourth as moderate risk of bias. Conclusions: Moderate, quality scientific evidence indicates a weak but significant relationship between smoking and the prevalence of RPLs in RFT. Smoking can be considered a negative prognostic factor for the outcome of root canal treatment. Endodontic providers should be aware of the relationship between smoking and persistent apical periodontitis, assessed as RPLs, in RFT.

## 1. Introduction

Apical periodontitis (AP) is an inflammatory reaction in the periradicular tissues, induced and maintained by bacterial infection of the root canal system [1]. The prevalence of AP is 0.6–20% for teeth [2,3]. AP is radiographically diagnosed by a disruption of the lamina dura and a radiolucent area encircling the root apex, namely, a radiolucent periapical lesion (RPL) [4]. Teeth with AP, when restorable, should be treated with root canal treatment (RCT) [5]. A key goal of RCT is to seal the apical third of the root canal, interrupting the passage of bacterial antigens from the pulp space to the periapical tissues. If this is not achieved, the root-filled tooth continues to show a radiolucent image around its apex, suffers from apical periodontitis [6,7,8], and presents—to some extent and severity—periapical inflammation [9]. RFT with AP in asymptomatic patients exhibited less pronounced and relatively smaller areas of inflammation [9]. Although radiographic signs of AP are found in 25 to 61% of asymptomatic RFT [3], not in all cases imply the failure of RCT. The healing after RCT may result in the formation of fibrous tissue composed of dense collagen fibers, few cells, and little or no inflammation, which may be regarded as scar tissue [10].

Apical periodontitis is not always the result of inadequate endodontic technique (including deficient aseptic control, missed canals, inadequate instrumentation, etc.) [11,12]. Sometimes, the systemic status of the patient, such as pro-inflammatory status or impaired immune response, can restrict periapical healing [13,14]. This could explain the association between diabetes and the failure of endodontic treatment, as recently demonstrated [15,16,17]. In short, the factors involved in the development of PAP and failure of RCT are many—it is difficult to assess the role that each of them plays. Knowing the possible influence of each one on the outcome of RCT can help to improve the information given to patients regarding the prognosis of RCT. In addition, it could help explain cases of patients in whom the failure of RCT is more frequent. This could be the case with tobacco smoking.

Habitual smoking, a systemic condition characterized by a pro-inflammatory status and impaired immune response and wound healing, has been associated with poor prognosis of periodontal disease, oral cancer, oral mucosa lesions, caries, and high failure rate of dental treatments [18,19]. Defensive and reparative responses of dental pulp are decreased in smokers [20], and tobacco smoking is a risk factor for periapical disease—AP being more prevalent in smokers [21,22,23,24,25,26], probably because of impaired bone healing [27]. A recent systematic review and meta-analysis concluded that tobacco smokers have a prevalence of periapical periodontitis and root canal treatments greater than 2.5 times the prevalence of non-smokers [28]. In addition, another study indicates that RCT is almost two times more prevalent in smokers, with a dose–response relationship [23]. Nevertheless, other studies have found no significant differences in the prevalence of AP and RCT between smokers and non-smoking subjects [13,29,30].

The possible effect of smoking on the outcome of endodontic treatment has been investigated in several epidemiological studies, with contradictory conclusions [31,32,33]. The primary objective of this study was to carry out a systematic review and meta-analysis investigating the possible association between smoking habits and the failure of RCT, the primary outcome measure being the prevalence of RPLs in RFT.

## 2. Methods

The protocol of this systematic review has been developed and registered in the PROSPERO database (PROSPERO 2020 CRD42020165279). The systematic review has been developed according to the Preferred Reporting Items for Systematic Reviews and Meta-Analyses (PRISMA) Guidelines [33].

### 2.1. Review Question

The clinical Population, Intervention, Comparison, and Outcome (PICO) question to be answered was as follows: in adult patients who have root filled teeth, does the presence or the absence of smoking habits affect the prevalence of RFT with RPLs? PICO (Population, Intervention, Comparison, and Outcome) schema for all the included studies to elaborate upon this research question were used to establish the eligibility criteria as follows:

Population: adult patients having root-filled teeth.

Intervention: presence of smoking habits; smoker.

Comparison: absence of smoking habits; non-smoker.

Outcome: prevalence of RFT with RPLs.

### 2.2. Inclusion and Exclusion Criteria

The inclusion criteria established were: (a) epidemiological studies published from January 1980 to June 2020; (b) studies comparing smoking patients with non-smoking subjects; (c) studies including RFT; (d) studies providing data on the prevalence of RFT with RPLs, both in smoking patients and in control non-smoking subjects. Exclusion criteria were defined as: (a) studies carried out in animals or in cell culture, and (b) studies reporting data only from smoking subjects. When there was no initial agreement among the reviewers, consensus was reached through dialogue.

### 2.3. Literature Search

Once the PICO question was established, the search strategy was designed [34,35]. Studies located in the search were selected according inclusion and exclusion criteria, quality evaluation, and data extraction and analysis. A literature search in MEDLINE/PubMed, Scopus, Web of Science, and Wiley Online Database was achieved, using the following Mesh terms and keywords: (tobacco OR smoking OR smoker) AND (endodontics OR periapical periodontitis OR periapical diseases OR apical periodontitis OR periradicular lesion OR periapical radiolucency OR radiolucent periapical lesion OR root canal treatment OR root canal preparation OR root canal therapy OR root filled teeth OR endodontically treated teeth) (Box 1).

Box 1. **MeSH and key words combinations used for the search strategy for the Population, Intervention, Comparison, and Outcome (PICO) question: In adult patients who have root filled teeth, does the absence or presence of smoking habits affect the prevalence of root filled teeth with radiolucent periapical lesions?**

((“tobacco”[MeSH Terms] OR “tobacco”[All Fields] OR “tobacco products”[MeSH Terms] OR (“tobacco”[All Fields] AND “products”[All Fields]) OR “tobacco products”[All Fields]) OR (“smoking”[MeSH Terms] OR “smoking”[All Fields]) OR (“smokers”[MeSH Terms] OR “smokers”[All Fields] OR “smoker”[All Fields])) AND ((“endodontics”[MeSH Terms] OR “endodontics”[All Fields]) OR (“periapical periodontitis”[MeSH Terms] OR (“periapical”[All Fields] AND “periodontitis”[All Fields]) OR “periapical periodontitis”[All Fields]) OR (“periapical diseases”[MeSH Terms] OR (“periapical”[All Fields] AND “diseases”[All Fields]) OR “periapical diseases”[All Fields]) OR (“periapical periodontitis”[MeSH Terms] OR (“periapical”[All Fields] AND “periodontitis”[All Fields]) OR “periapical periodontitis”[All Fields] OR (“apical”[All Fields] AND “periodontitis”[All Fields]) OR “apical periodontitis”[All Fields]) OR (Periradicular[All Fields] AND Lesion[All Fields]) OR (Periapical[All Fields] AND Radiolucency[All Fields]) OR (Radiolucent[All Fields] AND Periapical[All Fields] AND Lesion[All Fields]) OR ((“dental pulp cavity”[MeSH Terms] OR (“dental”[All Fields] AND “pulp”[All Fields] AND “cavity”[All Fields]) OR “dental pulp cavity”[All Fields] OR (“root”[All Fields] AND “canal”[All Fields]) OR “root canal”[All Fields]) AND (“therapy”[Subheading] OR “therapy”[All Fields] OR “treatment”[All Fields] OR “therapeutics”[MeSH Terms] OR “therapeutics”[All Fields])) OR (“root canal preparation”[MeSH Terms] OR (“root”[All Fields] AND “canal”[All Fields] AND “preparation”[All Fields]) OR “root canal preparation”[All Fields]) OR (“root canal therapy”[MeSH Terms] OR (“root”[All Fields] AND “canal”[All Fields] AND “therapy”[All Fields]) OR “root canal therapy”[All Fields]) OR ((“plant roots”[MeSH Terms] OR (“plant”[All Fields] AND “roots”[All Fields]) OR “plant roots”[All Fields] OR “root”[All Fields]) AND Filled[All Fields] AND (“tooth”[MeSH Terms] OR “tooth”[All Fields] OR “teeth”[All Fields])) OR (“tooth, nonvital”[MeSH Terms] OR (“tooth”[All Fields] AND “nonvital”[All Fields]) OR “nonvital tooth”[All Fields] OR (“endodontically”[All Fields] AND “treated”[All Fields] AND “teeth”[All Fields]) OR “endodontically treated teeth”[All Fields]))

A hand-search was also carried out in main endodontic journals (International Endodontic Journal, Journal of Endodontics, and Australian Endodontic Journal) and in the references of significant papers and reviews. The last search was made in June of 2020.

Electronic and manual searches provided the titles and abstracts of articles related to the aims of the studies, which were categorized by three independent researchers (D.C.-B., M.C.J.-S., and J.J.S.-E.) according to the inclusion and exclusion criteria. Articles selected were reviewed in full by five investigators (D.C.-B., J.M.-G., E.V.O., M.C.J.-S., and J.J.S.-E.).

### 2.4. Data Extraction

The methodology of selected studies was examined and main features were extracted and compiled, including: authors, date of publication, study design, subjects and sample size, main quantitative results and odds ratio values, and diagnoses of RPLs. Data extraction was performed by seven investigators (D.C.-B., J.M.-G., M.C.J.-S., E.V.O., B.S.-D., P.M.-M., and J.J.S.-E.). Disagreements were resolved by discussion among the six and reaching an agreement by majority.

### 2.5. Outcome Variables and Statistical Analysis

The primary outcome measure was the prevalence of RFT with RPL. Odds ratio (OR), with its 95% confidence interval (CI), was calculated in every selected study trying to measure the effect of the relationship between smoking habits and the outcome of RCT. A random-effect model meta-analysis, on the basis of the DerSimonian–Laird method, was performed to determine the pooled OR and its 95% CI. To determine the heterogeneity amongst trials, the Breslow–Day test (BDT) and the Higgins I2 test were employed, taking into account that substantial heterogeneity is considered if I2 test is higher than 50% [36]. To illustrate the homogeneity, L’Abbé plots [37] were used. To show the OR results, a forest plot [38] was used, along with the DerSimonian–Laird pooled estimate. Finally, a level of *p* = 0.05 was considered significant. The meta-analyses were calculated with the StatsDirects software (London, UK) [39].

### 2.6. Quality Evidence Assessment and Risk of Bias in Individual Studies

Quality evidence assessment and risk of bias in individual studies. The quality of evidence of the included studies was analyzed according to the guidelines provided by the Centre for Evidence-Based Medicine at Oxford [40]. The certainty in the evidence was assessed using the GRADE tool (GRADEpro GDT: GRADEpro Guideline Development Tool (Software)) available from gradepro.org: https://gdt.gradepro.org/app/handbook/handbook.html#h.rkkjpmwb6m6z [41]. The GRADE tool has five domains: risk of bias, inconsistency, imprecision, indirectness, and publication bias, which can be downgraded and reduce the quality of the evidence [42]. Articles were assessed independently by 5 reviewers (J.J.S.E., J.M.G., D.C.B., E.V.O., and M.C.J.S.) and cases of disagreements in the risk of bias were discussed until a consensus was achieved. The risk of bias of the included studies was assessed according to Cochrane Collaboration common scheme for bias and ROBINS-I tool [43], initially described to assess nonrandomized studies of interventions, but currently also available for observational designs (https://methods.cochrane.org/robins-i-tool).

## 3. Results

**Figure 1:**
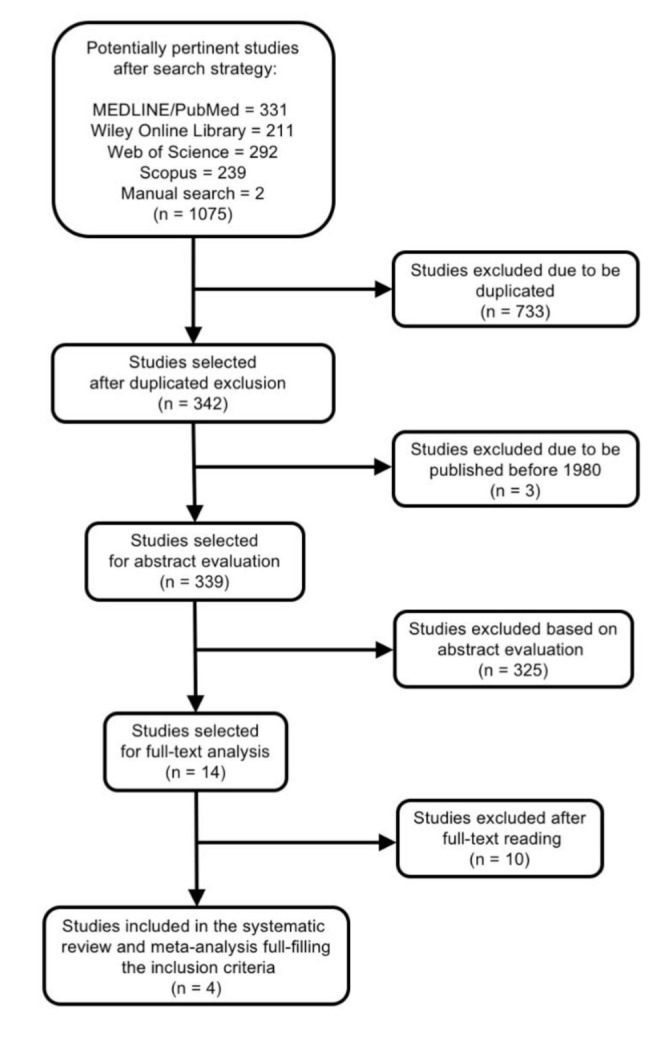
Selection process of the studies included in the systematic review and meta-analysis.

The search strategy is presented in Figure 1. After searching databases and hand-searching relevant bibliographies/papers, 1075 articles were recovered. Excluding duplicates articles (n = 733) and publications before 1980 (n = 3), 339 articles were checked to satisfy the selection criteria by title and abstract, declaring 14 articles for full text review. Among these, ten articles were excluded for the following reasons: four did not deal with the specific topic [25,26,44,45], five did not provide necessary data for meta-analysis [13,46,47,48,49], and one did not provide data on the frequency of AP at the root-filled teeth [50] (Table 1).

### 3.1. Study Characteristics

Four studies were finally included in the analysis: (1) Segura-Egea et al. (2008) [24]; (2) Segura-Egea et al. (2011) [51]; (3) Jansson (2015) [52]; (4) Sopińska and Bołtacz-Rzepkowska (2020) [53]. Study design, study sample, diagnosis of RPL, main results, and evidence level are summarized in Table 2.

### 3.2. Meta-Analysis

**Figure 2:**
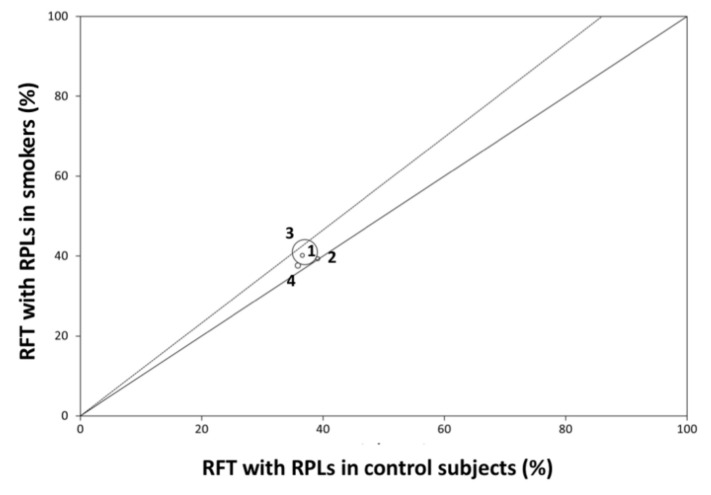
L’Abbé plot presenting the prevalence of root filled teeth (RFT) with radiolucent periapical lesions (RPLs) in each of the four studies in smoker patients and healthy controls. Circles of different sizes represent the weights of the sample of each study (1) Segura-Egea et al. (2008) [24]; (2) Segura-Egea et al. (2011) [51]; (3) Jansson (2015) [52]; (4) Sopińska and Bołtacz-Rzepkowska (2020) [53].

**Figure 3:**
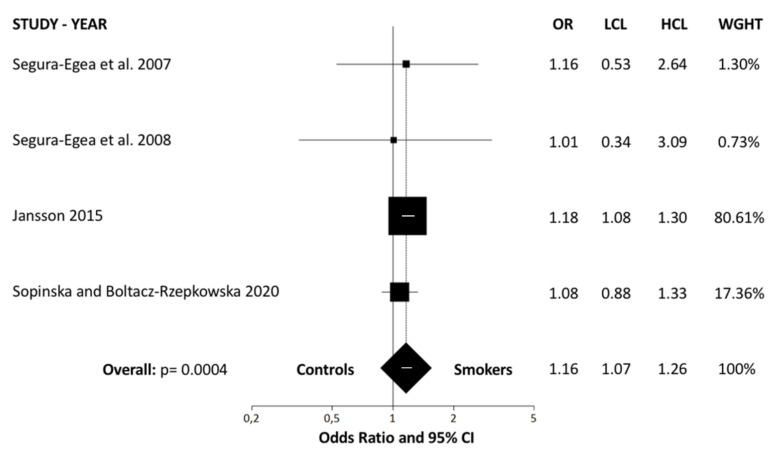
Forest plot of ORs and 95% confidence limits (CLs) for the comparison of smokers and healthy control subjects regarding the prevalence of root filled teeth (RFT) with radiolucent periapical lesions (RPLs). Overall estimate is based on data from the four studies. Black squares represent the point estimates of the OR and have areas proportional to study size. Lines represent 95% confidence intervals. The diamond shows the summary statistics for the four studies. The solid line indicates an OR of 1.0, and the dashed line indicates the overall odds ratio. OR: odds ratio; LCL: lower confidence level; UCL: upper confidence level.

Data from selected articles were analyzed and summarized in an evidence table containing the descriptive statistics and ORs calculated (Table 3). An overall OR greater than one implies that smoker patients present a higher prevalence of RFT with RPLs, compared to control subjects. Homogeneity among included studies was examined by Breslow–Day test (BDT)—the result was non-significant (Breslow–Day = 0.71; df = 3; *p* = 0.87) (Figure 2, L’Abbé plot). Moreover, heterogeneity test value (I² = 0%; 95% CI = 0% to 67.9%) was very low, so the proportion of variation through studies due to heterogeneity is not probable. The weights were calculated using a random effects model, allowing the study outcomes to vary in a normal distribution. Global OR was calculated using DerSimonian–Laird method with random effects, resulting in an OR = 1.16 (95% CI = 1.07–1.26; *p* = 0.0004). The ORs for each study and the pooled OR from the meta-analysis are shown in a forest plot (Figure 3). The results of the meta-analysis indicate that the prevalence of RFT associated with RPLs in smoking patients differs significantly from the prevalence in control subjects.

### 3.3. Interpretation and Assessment of the Included Studies

**Figure 4:**
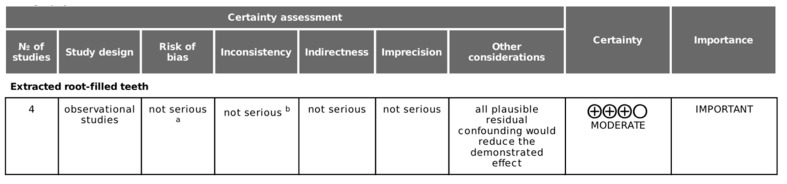
GRADE Working Group grades of evidence: Smoking habits and the prevalence of radiolucent periapical lesions in root-filled teeth. Explanations: a. Detailed in Figure 5: Risk of bias summary, b. I^2^ = 0%. High certainty: the authors have a lot of confidence that the true effect is similar to the estimated effect. Moderate certainty: the authors believe that the true effect is probably close to the estimated effect. Low certainty: the true effect might be markedly different from the estimated effect. Very low certainty: the true effect is probably markedly different from the estimated effect.

The four studies included in the meta-analysis (Figure 4) were cross-sectional, all published between 2008 and 2020. The data obtained from the studies, 9257 RFT, 4465 in non-smoker control subjects and 4792 in smoker patients, were compiled.

The prevalence of apical periodontitis amongst smokers was investigated in the study of Segura-Egea et al. [24], who concluded there is a significantly association between smoking and increased prevalence of AP and higher frequency of RCT. However, although the presence of AP in RFT was higher in smoker patients (71%) with respect to non-smokers (55%), the difference was not significant (OR = 1.16; 95% CI = 0.53–2.64; *p* = 0.69).

Another study by Segura-Egea et al. [51], carried out in hypertensive patients, analyzed the interrelationship between endodontic variables and smoking habits. Although significantly higher prevalences of AP and RCT were found in smokers, the frequency of RFT with AP in smoker hypertensive patients (64.9%) was not higher than in non-smoker patients (64.3%) (OR = 1.01; 95% CI = 0.34–3.09; *p* = 0.99).

The study of Jansson et al. [52] aimed to investigate the relationship between the presence of AP in RFT and marginal bone loss. The results showed a significant correlation between smoking and the prevalence of RFT with AP, this prevalence being 41% in smokers and 37% in non-smoker subjects (OR = 1.18; 95% CI = 1.08–1.30; *p* = 0.00045). However, multiple regression analysis indicated that the relative frequency of RFT with AP was significantly associated with more marginal bone loss, irrespective of age, number of remaining teeth, relative frequency of root-filled teeth, and smoking habits.

Finally, the recent study conducted by Sopińska and Bołtacz-Rzepkowska [53] aimed to evaluate the influence of smoking on the prevalence of AP in the population of the Łódź region, Poland. Results show no difference in the frequency of RFT with AP between smokers (37.6%) and control subjects (35.8%) (OR = 1.08; 95% CI = 0.88–1.33; *p* = 0.451).

### 3.4. Quality Evidence and Risk of Bias Assessment

**Figure 5:**
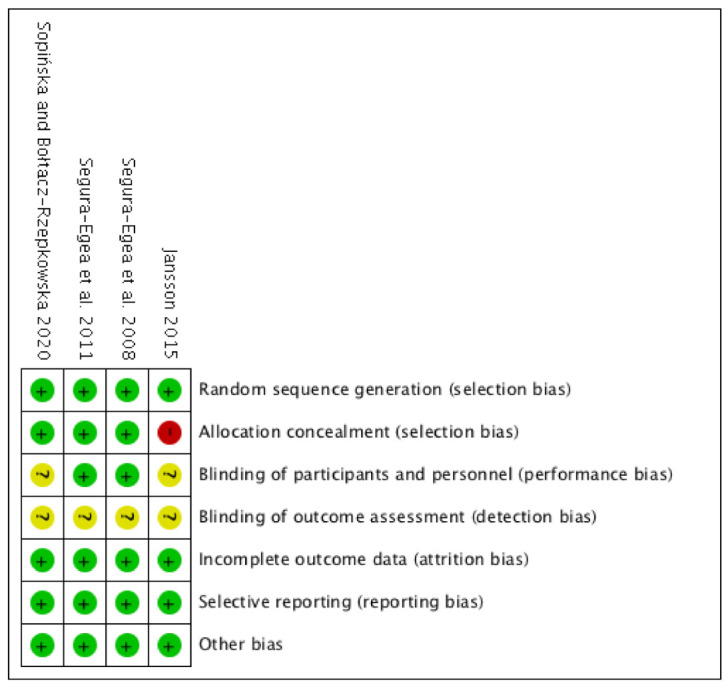
Risk of bias summary of studies included according to the Cochrane Collaboration’s tool for assessing risk of bias. + Low risk of bias. − High risk of bias. ? Unclear risk of bias.

The scores for the methodological quality of the articles included in this systematic review are given in Table 2. The Centre for Evidence-Based Medicine at Oxford [40] scores for the studies were low, all of them rated with level 4. The GRADE tool demonstrated a moderate quality of the evidence for the included studies (Figure 4). According to ROBINS-I tool, from the four included studies, three were classified as low risk of bias, with only one or two domains as unclear risk of bias (Segura-Egea et al. 2008, Segura-Egea et al. 2011, Sopińska and Bołtacz-Rzepkowska 2020), and the other was classified as moderate risk of bias with one domain as high and two domains as unclear risk of bias (Jansson 2015) (Figure 5).

## 4. Discussion

This study aimed to analyze the possible link between smoking habits and the outcome of RCT. Therefore, a systematic review and meta-analysis has been conducted, including the available evidence about the prevalence of RFT with RPLs. After the literature search, four studies were included in the final analysis, all analyzing the prevalence of RFT with RPLs [24,48,49,50,51,52,53] in both smokers and non-smoker subjects. The four studies were cross-sectional studies.

The four included studies analyzed 9257 root-filled teeth, 4465 in non-smokers and 4792 in smoker patients. The random effects model was used to calculate overall ORs, allowing the study outcome to vary in a normal distribution. The heterogeneity value in the primary outcome measure was null (0%), suggesting that there is no variability between the studies. For the association between smoking habits and the prevalence of RFT with RPL, the DerSimonian–Laird method reported an overall OR = 1.16, statistically significant (*p* = 0.0004). Thus, the results of the present meta-analysis suggest that smoking habits increase the risk of failure of RCT and the prevalence of RPLs in RFT. The RFT of a smoking patient are 1.16 times more likely to have radiolucent periapical lesions compared to non-smoking subjects.

Smoking has been recognized as an important risk factor for cardiovascular disease [54] and periodontal disease, increasing inflammation of the periodontium and marginal bone loss [52,55,56,57]. Moreover, a significant association between AP and smoking habits has been described [21,22,25,42]. The results of the systematic review carried out by Aminoshariae et al. [58] analyzing the association between smoking and the prevalence of apical periodontitis, suggest that smoking was associated with the prevalence of AP in cross-sectional studies and case control studies. A systematic review with meta-analysis has just been published reporting a significant association between smoking and the loss of root-filled teeth [59]. However, the associations between smoking and the prevalence of AP have not been investigated so far by meta-analysis. The results of the present study fill this knowledge gap.

Smoking could influence the outcome of RCT, probably impairing periapical status of RFT, maintaining the periapical bone destruction, and decreasing the healing after RCT [14]. The effect of tobacco smoking on periapical disease has biological plausibility and can be explained by several biological mechanisms [14]. Smoking habits provoke impaired functions of leukocytes, macrophages, and T-cell lymphocytes, with decreased levels of antibodies [60], and increased levels of pro-inflammatory mediators, such as IL-6, TNF-α, and C-reactive protein [61,62,63,64]. Smoking also causes morphological and functional alterations of the microcirculation. Increased carboxyhemoglobin levels and oxidative stress injure microvascular function, decreasing the oxygen supply and nutrient delivery [65]. It can be hypothesized that inflamed periapical tissues in smokers could experience restrictions in nutrients and oxygen supply [14]. On the other hand, tobacco smoking has been shown to cause delay fibroblast migration to the wound area and fibroblast dysfunction [66]. Finally, a local and direct pro-inflammatory effect of smoking on periapical tissues has been demonstrated. In smokers with granuloma due to AP, the products of lipid peroxidation, as 8-iso-PGF(2a) and products of the LOX-pathway, were increased at the expense of cyclooxygenase products [67]. Therefore, smoking decreases bone healing and tissue response, due to high stimulation of osteoclastic cells and reduced angiogenesis [29,68].

The results of the present systematic review and meta-analysis should be valued with caution. According to the Centre for Evidence-Based Medicine at Oxford [40], the quality level of the four included studies is low. This could be considered a limitation of the study. However, the ROBINS-I tool classified as low risk of bias three of the included studies, and the GRADE tool demonstrated a moderate strength of evidence, indicating that the true effect is probably comparable to the estimated effect. Prospective studies comparing the outcome of endodontic treatment in smokers and non-smokers should be carried out, taking into account the amount of tobacco smoked and the time during which the patients have been smokers.

The present systematic review has some limitations. The included studies considered that a radiolucency associated with a RFT was a sign of AP. However, in a cross-sectional study it is not possible to know if the RPL is disease or healing in progress. Furthermore, the healing after RCT may result in the formation of scar tissue [10]. The method to assess the periapical status is an important factor that should be taken into account, and it is different in each of the studies included in the review. Moreover, the included studies used conventional radiographs or panoramic radiographs for the diagnosis of AP. Future studies evaluating periapical lesions should include three-dimensional diagnostic methods, such as CBCT. CBCT allows radiological signs to be identified with greater sensitivity, so it better evaluates changes in hard tissue and periapical bone repair [69,70].

## 5. Conclusions

Available scientific evidence indicates a weak but significant relationship between smoking and apical periodontitis in root filled teeth. However, the quality of the evidence is moderate. Better-designed longitudinal studies are necessary to define with accuracy the impact of smoking on the outcome of RCT. Meanwhile, habitual smoking should be considered a preoperative risk factor for RCT, since it reduces or limits its success, increasing the frequency of periapical lesions in endodontically treated teeth.

## Figures and Tables

**Table 1:** Studies excluded in the systematic review of association between smoking habits and the prevalence of radiolucent periapical lesions (RPLs) in root-filled teeth (RFT). Excluded reason, authors, and year of these studies.

Excluded Reason	Authors	Year/Reference
Not specific topic	1. Kirkevang et al.	2007/[46]
	2. López-López et al.	2012/[25]
	3. Oginni et al.	2015/[26]
	4. Olcay et al.	2018/[44]
Not provide necessary data to meta-analysis	5. Marending et al.	2005/[13]
	6. Peršić Bukmir et al.	2016/[49]
	7. Al-Nazhan et al.	2017/[48]
	8. Pirani et al.	2018/[46]
	9. Alghofaily et al.	2018/[47]
Not provide data of RPLs in RFT	10. Doyle et al.	2007/[50]

**Table 2:** Studies about smoking habits and the prevalence of root filled teeth (RFT) with radiolucent periapical lesions (RPLs) included in the systematic review. Study design, subjects and sample size, diagnosis of RPL, main results, and evidence level.

Authors/Year/Ref.	Study Design	Subjects	Diagnosis of RPLs	Main Results	Evidence Level
Segura-Egea et al. 2008 [24]	Cross-sectional	Controls: 71 Smokers: 109	14 periapical radiographs Paralleling technique Periapical Index (PAI)	No association; *p* = 0.6868	4
Segura-Egea et al. 2011 [51]	Cross-sectional	Controls: 50 Smokers: 50	14 periapical radiographs Paralleling technique Periapical Index (PAI)	No association; *p* = 0.9857	4
Jansson 2015 [52]	Cross-sectional	Controls: 576 Smokers: 576	18 periapical radiographs Widened periodontal space and not visible lamina dura	Association; *p* = 0.00045	4
Sopińska and Bołtacz-Rzepkowska 2020 [53]	Cross-sectional	Controls: 317 Smokers: 386	Panoramic radiograph Twice width periodontal space or demarcated with osteosclerotic border	No association; *p* = 0.451	4

**Table 3:** Studies about smoking habits and the prevalence of root filled teeth (RFT) with radiolucent periapical lesions (RPLs). Results extracted and compiled, descriptive statistics, and calculated odds ratios.

Authors/Ref.	No. RFT	Control Subjects		Smoker Patients		Odds Ratio (95% CI)	p
		RFT*RPL/Total RFT	RFT*RPL (%)	RFT*RPL/Total RFT	RFT*RPL (%)		
1. Segura-Egea et al. 2008 [24]	153	15/41	37%	45/112	40%	1.16 (0.53–2.64)	*p* = 0.69
2. Segura-Egea et al. 2011 [51]	84	9/23	39%	24/61	39%	1.01 (0.34–3.09)	*p* = 0.99
3. Jansson 2015 [52]	7368	1363/3684	37%	1510/3684	41%	1.18 (1.08–1.30)	*p* = 0.0005
4. Sopińska and Bołtacz-Rzepkowska 2020 [53]	1652	257/717	36%	352/935	38%	1.08 (0.88–1.33)	*p* = 0.451
**Overall**	9257	1644/4465	36.82%	1931/4792	40.30%	1.16 * (1.07–1.26)	***p*** **= 0.0004**

* DerSimonian–Laird variance formula: Chi² = 12.338298, *p* = 0.0004.

## Supplementary Materials

The following are available online at https://www.mdpi.com/2077-0383/9/11/3506/s1, Prisma Checklist.
